# Remimazolam-Based Anesthesia in Patients with Heart Failure Due to Mitral Regurgitation and Low Left Ventricular Function: A Case Series

**DOI:** 10.3390/medicina59122136

**Published:** 2023-12-08

**Authors:** Atsuhiro Kitaura, Hiroatsu Sakamoto, Shinichi Hamasaki, Shota Tsukimoto, Yasufumi Nakajima

**Affiliations:** Department of Anesthesiology, Kindai University Faculty of Medicine, Osaka 586-0001, Japanshtsukimoto@gmail.com (S.T.); nakajima@med.kindai.ac.jp (Y.N.)

**Keywords:** remimazolam, MitraClip, mitral regurgitation, heart failure, anesthesia

## Abstract

*Background and Objectives*: Remimazolam is a new ultrashort-acting benzodiazepine anesthetic. Remimazolam appears to be useful in patients with severe valvular disease because of its minimal cardiovascular impact. In this retrospective case series study, we assessed the efficacy and safety of remimazolam for maintaining hemodynamic stability during anesthetic induction and maintenance. *Cases*: MitraClip was performed on 18 cases with severe mitral regurgitation with low left ventricular function who presented with heart failure, and remimazolam was administered for general anesthesia with induction (12 mg/kg/h) and maintenance (1 mg/kg/h). The impact of remimazolam on the hemodynamics at anesthetic induction and during anesthetic maintenance was investigated retrospectively using electronic medical records. Blood pressure decreased significantly during anesthetic induction with remimazolam (78.5 [72, 81.25] and 66.1 [62.2, 74.2], median [IQR], *p* = 0.0001), but only mildly, by about 10 mmHg. There was no significant change in the cardiac index (2.0 [1.8, 2.4] vs. 1.9 [1.8, 2.3], *p* = 0.57642) or pulse rate (73.5 ± 8.85 vs. 74.7 ± 11.7, mean ± SD, *p* = 0.0876) during anesthetic induction with remimazolam. All patients underwent MitraClip without major hemodynamic concerns, with no or small increases in inotropes. *Conclusions:* Remimazolam may be used safely in patients with severe mitral regurgitation and low left ventricular function presenting with heart failure.

## 1. Introduction

Intravenous anesthetics are commonly used general anesthetics. Intravenous anesthetics have the known advantages of less air pollution [1], less excitement during induction and awakening [2], and less postoperative nausea and vomiting (PONV) [3]. Propofol, currently the most frequently used intravenous anesthetic, provides rapid anesthetic effects. However, propofol is associated with relatively strong cardiovascular depression and vascular pain [4]. Midazolam, a short-acting benzodiazepine currently in common use, has less of a cardiovascular impact than propofol, but its longer duration of action requires more time for anesthesia induction and awakening, leading to a burden on healthcare professionals [5]. Remimazolam, an ultrashort-acting benzodiazepine, became available in Japan in 2020 [6]. Remimazolam acts on the benzodiazepine binding site of the gamma amino butyric acid (GABA) A receptor and facilitates the action of GABA, a major inhibitory neurotransmitter, on the receptor [7]. Remimazolam is quickly metabolized in the liver and has almost no cumulative effect [8]. In addition, it is reversible since an antagonist with nearly the same half-life is available [6]. Remimazolam provides rapid anesthetic effects similar to those of propofol, with the safety of reliable antagonism and minimal hemodynamic impact, the hallmark of benzodiazepine [9,10]. The safety of remimazolam has already been proven in ASA-PS:3 patients [10]. Therefore, we also use remimazolam in high-risk patients. However, the safety of remimazolam in ASA-PS:4 patients has not yet been proven, and case reports are scarce [11,12,13,14,15]. Therefore, we hypothesized that remimazolam could be safely used not only in ASA-PS:3 patients but also in ASA-PS:4 patients. We present a retrospective case series of patients with severe mitral regurgitation and low cardiac function who underwent MitraClip at our hospital with a review of the literature, with the aim of evaluating the safety of remimazolam in ASA-PS:4 patients.

## 2. Case Presentations

Consent was obtained in writing from all patients before the study was conducted. This study is a retrospective case series study using electronic medical records. Among all MitraClip cases performed at our hospital after 2021, only patients with severe mitral regurgitation, preoperative low left ventricular function (left ventricular ejection fraction; LVEF < 0.4), and heart failure (New York Heart Association Classification; NYHA ≥ 2) were selected from the electronic medical records. For all extracted patients, the electronic anesthesia records were used to extract the patient’s preoperative information (age, sex, height, weight, body mass index, NYHA, LVEF, MR severity, Euro 2 score, preoperative inotrope, estimated glomerular filtration rate (eGFR), brain natriuretic peptide (BNP); comorbidities); anesthesia time; operative time; infusion volume; time required for induction with remimazolam; circulatory parameters such as blood pressure, pulse rate (PR), cardiac index (CI), and stroke volume valuation (SVV); dose of remimazolam; noradrenaline; and use of inotropic drugs such as dobutamine. Among them, the induction dose of remimazolam, blood pressure, cardiac index, and pulse rate before and after induction were analyzed to evaluate the impact of induction with remimazolam on the hemodynamics. In addition, to evaluate the intraoperative hemodynamics, the maintenance dose of remimazolam and the intraoperative inotropes dose were analyzed. 

### Anesthesia

All patients were anesthetized according to the same in-hospital protocol. No premedication was administered. Angiotensin-converting enzyme inhibitors, angiotensin 2 receptor blockers, and oral diabetes medications were discontinued on the day of surgery. On arrival at the operation room, a standard monitor for non-invasive blood pressure (NIBP), continuous electrocardiogram (ECG), pulse oximeter (S_P_O_2_), and bispectral index (BIS; Medtronic Co., Minneapolis, MN, USA) were attached. Inotropic drugs were continued if they were administered preoperatively. Prior to the induction of anesthesia, an arterial blood gas analysis was performed under local anesthesia with an arterial pressure line secured. A Flo Trac sensor (Edwards life-science Co., Irvine, CA, USA) was connected to the arterial pressure line and to start the measurement. General anesthesia was maintained using the anesthesia method with remimazolam and remifentanil. Remimazolam was administered as a loading dose at 12 mg/kg/min and maintained at 1 mg/kg/h after loss of consciousness. No other drugs were administered during remimazolam-loading. The time required to fall asleep was measured. After loss of consciousness, tracheal intubation was performed with the administration of 0.05 mg remifentanil and 0.6 mg/kg rocuronium. A central venous catheter was secured in the right internal jugular vein, and a transesophageal echocardiography (TEE) probe was inserted. Respiration during anesthesia was managed with mechanical ventilation. Intraoperative analgesia was administered with continuous infusion of 0.05 µg/kg/min remifentanil. Additionally, 15 mg/kg acetaminophen was administered intravenously for postoperative analgesia for all cases. After completion of femoral artery cannulation, 100 units/kg of heparin was administered to prolong activated clotting time to at least 250 s. Dexamethasone sodium phosphate (6.6 mg) was administered intravenously for all patients to prevent postoperative nausea and vomiting. Anesthetic depth was maintained in the range of 40–70 on a BIS monitor. In case of deviations, the remimazolam dose was adjusted between 1 and 2 mg/kg/h. During the maintenance of anesthesia, noradrenaline administration was initiated if the mean blood pressure (MAP) fell below 65 mmHg. Increased doses of inotrope (dobutamine or adrenaline) were allowed for the decline in cardiac function (cardiac index < 2.2 L/min/m^2^, ScvO_2_ < 65%) associated with the surgical maneuver. The choice of inotropic drugs depended on the anesthesiologist. All anesthetics were discontinued upon completion of the postoperative TEE study. After surgery and postoperative X-rays, the patient’s consciousness was checked. Flumazenil (0.5 mg) was administered to all patients. The patient was transferred to the Post-anesthesia Care Unit while continuing to recover consciousness. The day after the surgery, the patients were interviewed about awareness during anesthesia.

## 3. Results

This case series included 18 adult patients (men: 13, women: 5) who underwent a MitraClip procedure under general anesthesia. All cases included in this series were patients with severe mitral regurgitation with heart failure (ASA-PS:4, age: 74.7 [67.7, 81.25], NYHA: 3.5 [3,4], LVEF: 0.3 [0.26, 0.32]). The percentage of patients dependent on inotropic drugs was 59%. The preoperative status of the patients is shown in Table 1. 

**Figure 1:**
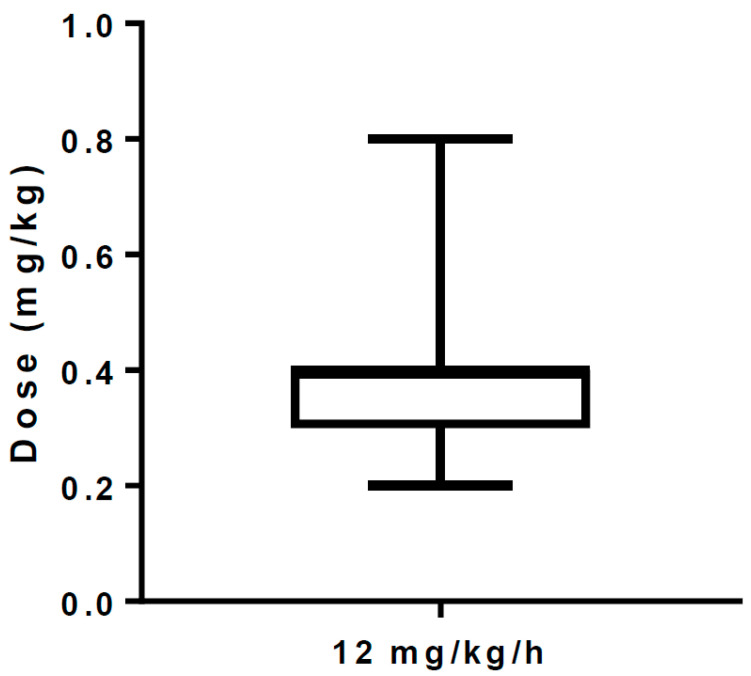
The induction dose of remimazolam in this study. The mean induction dose was also 0.35 mg/kg, which was higher than the 0.1 mg/kg at which all the elderly in the phase I clinical trial fell asleep. There was also a large variation among cases. Whiskers indicate maximum and minimum values.

**Figure 2:**
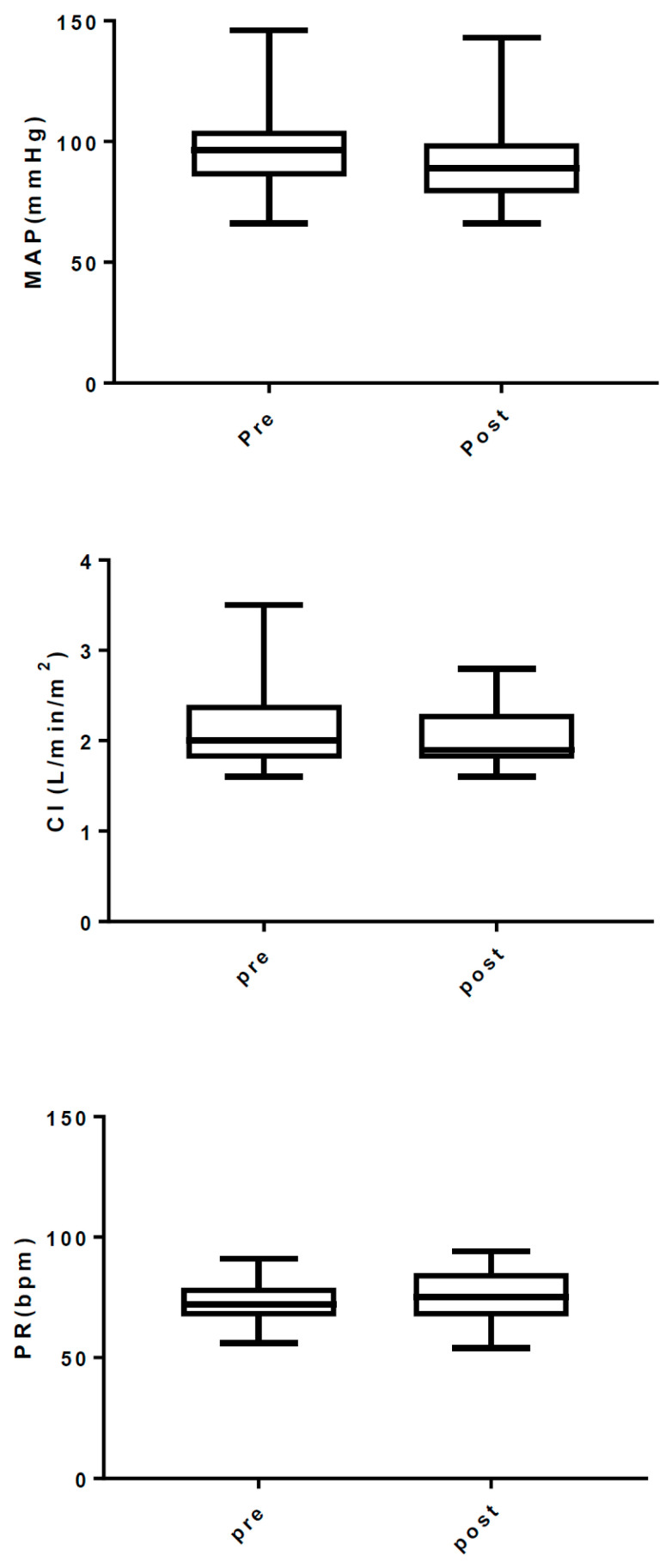
The changes in cardiovascular parameters before and after induction with remimazolam. MAP decreased significantly after induction, but the change was relatively small. CI and PR were maintained and did not differ significantly. MAP: mean arterial pressure, CI: cardiac index, PR: pulse rate. Whiskers indicate maximum and minimum values.

Anesthetic effects were observed in all cases. No patient required additional doses of anesthesia due to a tendency to arousal. The induction dose of remimazolam was 0.35 ± 0.13 mg/kg (mean ± SD) (Figure 1). The changes in the cardiovascular parameters before and after induction of anesthesia with remimazolam at 12 mg/kg/h are shown in Figure 2. The MAP before and after induction was 78.5 [72, 81.25] and 66.1 [62.2, 74.2] (median [IQR]). MAP decreased significantly (paired t test, one tailed, *p* = 0.0001), but only mildly, by about 10 mmHg. CI (2.0 [1.8, 2.4] vs. 1.9 [1.8, 2.3]) (paired t-test, one-tailed, *p* = 0.5762) and pulse rate (73.5 ± 8.85 vs. 74.7 ± 11.7) (paired t-test, one-tailed, *p* = 0.0876) did not change significantly.

Data on the overall duration of anesthesia are presented in Table 2. The anesthesia time was 148 [121, 179] minutes; the operation time was 79 [59, 125] minutes. The volume of infusion during anesthesia was 1210 [945, 1741] mL; the urine volume was 190 [110, 455] mL. The maximum dose of noradrenaline was 0.03 [0.03, 0.06] µg/kg/min. Only dobutamine was used in these cases as the inotrope. An increase or new initiation of inotropic drugs during anesthesia was observed in three cases, with an average increase in dose of about 2 µg/kg/min. In all cases, there were no cases of severe circulatory complications such as cardiac arrest or fatal arrhythmia during surgery. 

All patients awoke promptly after flumazenil administration. No re-sedation or postoperative respiratory arrest was observed. No patients had intraoperative memories of awakening.

## 4. Discussion

Remimazolam is a new benzodiazepine anesthetic that has been reported to have minimal effects on cardiac function [9]. In addition, its safety in patients with ASA-PS3 has already been reported [10]. Based on this, one would assume that the affinity for high-risk patients would be good; unfortunately, evidence in patients who fall into the ASA-PS:4 category is scarce. There are no randomized controlled trials in ASA-PS4 patients and, to the best of our knowledge, only a few case reports [11,12,13,14,15].

In this study, the blood pressure drop during induction with remimazolam was relatively mild, with a mean value of about 12 mmHg. PR was not significantly changed. Maintaining PR was considered advantageous for the patients with MR and low LVEF. Thus, the present results suggest that remimazolam can be used for induction of general anesthesia in patients with low cardiac function and severe mitral regurgitation equivalent to ASA-PS4, with mild hemodynamic variability being acceptable. The average induction dose of remimazolam in this study was 0.35 mg/kg. This induction dose was considered excessive. The remimazolam loading dose in this study was 12 mg/kg/h in accordance with the package insert in Japan. However, this dosing regimen has been reported to have a tendency to administer more remimazolam than necessary [8,9]. In a phase I clinical study, remimazolam was reported to induce sleep at a dose of 0.1 mg/kg in elderly patients and 0.2 mg/kg in healthy adults [8]. In the Japanese clinical trials, special care was taken to reduce the dead space in the remimazolam administration route as much as possible and to assign dedicated staff to accurately assess loss of consciousness [9]. Nevertheless, the mean cumulative dose of remimazolam required for induction was 0.29 mg/kg in the 12 mg/kg/h [9]. In actual clinical practice, however, such considerations are almost impossible. It is also known that the increase in effect site concentration is slower in patients with heart failure; as a result, there may be limitations to titration using the phenotype of sleep onset as an indicator. Therefore, decreasing the dosage rate or giving a single dose of about 0.1 mg/kg could have reduced the dosage more and may have lessened the effects on circulatory dynamics. Therefore, there is room for improvement in the method of administering remimazolam at induction.

The results of this study show that noradrenaline use was also relatively low, about 0.03 [0.03, 0.06] µg/kg/min. And, only three cases required increased doses of dobutamine due to general anesthesia. Inotropic drug use was also increased by about 2 µg/kg/min, and, except during temporary circulatory fluctuations due to the surgical procedure, circulatory dynamics were mostly stable. In addition, no intraoperative circulatory collapse or anesthetic complications were observed in this study. Thus, the results of this study suggest that remimazolam may be safe enough to use in ASA-PS4 patients with low left ventricular function and severe mitral regurgitation. 

The limitations of this study were as follows: This study involved a case series with a limited number of cases at a single institution. In addition, the data obtained were limited due to the retrospective nature of the study. Therefore, there were limitations in the conclusions that could be drawn from this study. Therefore, a prospective randomized controlled trial is needed to prove the safety of remimazolam. In addition, there is room for improvement in the method of remimazolam administration. Appropriate remimazolam dosing methods could lead to a greater emphasis on the benefits of remimazolam.

## 5. Conclusions

Remimazolam provided sufficiently stable hemodynamics during anesthesia in the present study. The blood pressure drop during induction with remimazolam was also relatively mild. The results of the present study suggested that remimazolam may be safe enough to use in patients with low left ventricular function and severe mitral regurgitation.

## Figures and Tables

**Table 1:** Demographic data of patients.

Variables	Age, Years	Sex	Height (cm)	Weight (kg)	NYHA	LVEF	MR	Euro Score	Preoperative Dobutamine (µg/kg/min)	eGFR	BNP	Comorbidities
Patient 1	82	Male	165	81	2	0.30	3	37.67	3	28	310.9	ICM
Patient 2	44	Male	155	42	3	0.40	3	2.07	2	4	195.4	MELAS
Patient 3	69	Male	171	55	4	0.30	4	3.09	4	55	269.4	DCM
Patient 4	81	Male	171	52	2	0.26	4	7.85	4	40	185.8	DCM
Patient 5	93	Male	171	57	3	0.27	4	9.67	3	38	803.6	ICM
Patient 6	67	Male	167	61	3	0.27	3	3.00	0	82	333.0	DCM
Patient 7	76	Male	160	64	3	0.22	4	16.73	3	28	69.4	DCM
Patient 8	68	Male	168	71	3	0.30	4	19.80	3	22	1335.8	ICM
Patient 9	88	Female	145	39	4	0.39	4	6.77	1	39	1939.5	unclear
Patient 10	74	Male	159	53	4	0.18	4	18.57	4	63	910.2	DCM s/o
Patient 11	81	Female	139	56	2	0.32	4	9.39	0	25	1765.4	ICM
Patient 12	56	Male	169	98	4	0.27	4	19.03	2	28	1047.0	DCM s/o
Patient 13	73	Female	155	56	3	0.30	4	5.93	0	50	956.8	ICM
Patient 14	81	Male	163	55	4	0.30	3	6.30	3	47	533.5	DCM
Patient 15	84	Female	153	40	4	0.23	4	29.05	0	46	179.0	ICM
Patient 16	81	Male	164	58	3	0.33	4	4.23	0	10	3036.0	ICM
Patient 17	81	Female	146	46	3	0.35	4	8.67	0	41	757.5	ICM
Patient 18	67	Male	162	64	4	0.31	4	15.99	0	33	1220.1	DCM
Total	74.7 (67.7, 81.25)		160 (154, 168)	58.2 (50.4, 64)	3.5 (3, 4)	0.3 (0.26, 0.32)	4 (3.7, 4)	9 (5.5, 18.6)		39 (27.2, 47.7)	781 (250, 1249)	

NYHA: New York Heart Association Functional Classification, LVEF: left ventricular ejection function, MR: mitral regurgitation, Euro score: Euro 2 score for cardiac surgery risk assessment, eGFR: estimated glomerular filtration rate, BNP: brain natriuretic peptide, ICM: ischemic cardiomyopathy, MELAS: mitochondrial myopathy, Encephalopathy, Lactic Acidosis, Stroke-like episodes, DCM: dilated cardiomyopathy. s/o: suspected, Data are expressed as median (IQR).

**Table 2:** Perioperative data of patients.

	Remimazolam Induction Dose, µg/kg	Remimazolam Maintenance Dose, mg/kg/h	Anesthesia Time, min	Operation Time, min	Maximum Dose of Noradrenaline, µg/kg/min	Maximum Dose of Dobutamine, µg/kg/min	Increase Dosage of Dobutamine, µg/kg/min
Patient 1	0.4	1	269	196	0.05	5	2
Patient 2	0.6	1	256	187	0.02	2	No
Patient 3	0.6	1	151	79	0.06	4	No
Patient 4	0.2	1	286	222	0.06	4	No
Patient 5	0.4	1	104	40	0.03	3	No
Patient 6	0.2	1	165	119	0.03	0	No
Patient 7	0.2	1	198	146	0.03	3	No
Patient 8	0.2	1	173	80	0.03	3	No
Patient 9	0.5	1	106	39	0.06	1	No
Patient 10	0.2	1	137	65	0.06	6	2
Patient 11	0.4	1	103	43	0.1	0	No
Patient 12	0.4	1	155	78	0.03	3	1
Patient 13	0.4	1	102	51	0.06	0	No
Patient 14	0.4	1	146	89	0.02	3	No
Patient 15	0.3	1	138	87	0.03	0	No
Patient 16	0.4	1	155	90	0.03	0	No
Patient 17	0.4	1	126	73	0.05	0	No
Patient 18	0.1	1	127	55	0.03	0	No
Total	0.35 ± 0.13		148 (121, 179)	80 (54, 125)	0.03 (0.03, 0.06)	2.5 (0, 3.5)	

Mean ± S.D. or median (IQR).

## Data Availability

The datasets of the current study are available from the corresponding author upon reasonable request.
